# Electrical Control of Photoluminescence in 2D Semiconductors Coupled to Plasmonic Lattices

**DOI:** 10.1021/acsnano.4c15459

**Published:** 2025-01-20

**Authors:** Antti J. Moilanen, Moritz Cavigelli, Takashi Taniguchi, Kenji Watanabe, Lukas Novotny

**Affiliations:** † Photonics Laboratory, ETH Zürich, CH-8093 Zürich, Switzerland; ‡ Research Center for Materials Nanoarchitectonics, National Institute for Materials Science, 1-1 Namiki, Tsukuba 305-0044, Japan; § Research Center for Electronic and Optical Materials, National Institute for Materials Science, 1-1 Namiki, Tsukuba 305-0044, Japan

**Keywords:** plasmonics, nanophotonics, surface lattice resonance, transition metal dichalcogenides, 2D semiconductors

## Abstract

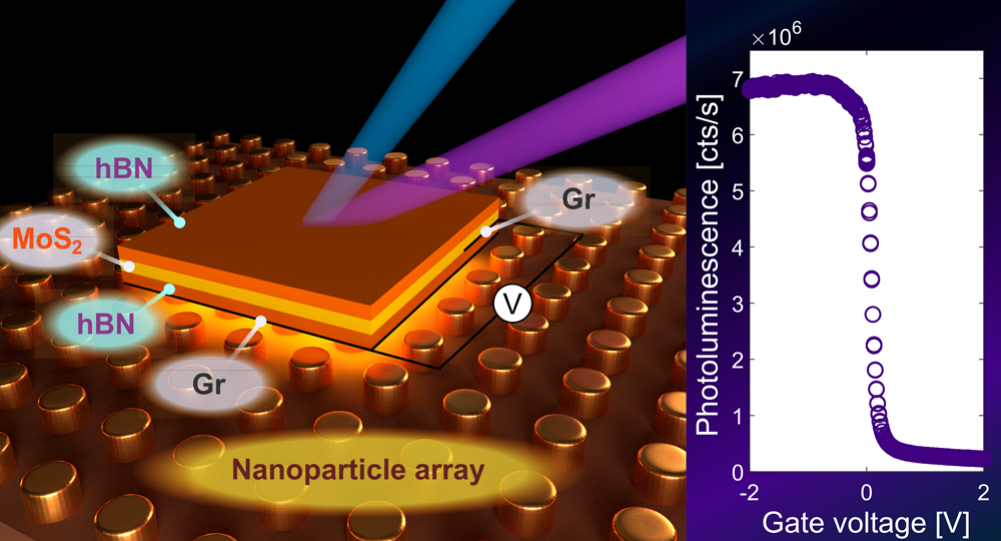

Integrating two-dimensional (2D) semiconductors into nanophotonic structures provides a versatile platform for advanced optoelectronic devices. A key challenge in realizing these systems is to achieve control over light emission from these materials. In this work, we demonstrate the modulation of photoluminescence (PL) in transition metal dichalcogenides (TMDs) coupled to surface lattice resonances in metal nanoparticle arrays. We show that both the intensity and the emission angle of light can be tuned by adjusting the lattice parameters. By applying gate electrodes to electrostatically dope the TMDs coupled to plasmonic lattices, we achieve PL intensity switching over 2 orders of magnitude with a low applied voltage. Our results represent an important step toward electrically powered and electrically tunable light sources based on 2D semiconductors.

Semiconducting transition metal dichalcogenides (TMDs) have attracted significant interest over the past decade due to their exceptional optical and electrical properties.^1,2^ Unlike their bulk counterparts, single-layer TMDs possess a direct optical bandgap, making them ideal active materials for nanoscale optoelectronics.^3,4^ Similar to other two-dimensional (2D) materials, such as semimetallic graphene and insulating hexagonal boron nitride (hBN), the layers in TMDs are held together by weak electrostatic van der Waals forces. This enables the exfoliation of pristine monolayers from bulk crystals and facilitates the stacking of different 2D materials without the typical issues of lattice mismatch or dangling bonds, which often limit material combinations in conventional complementary metal-oxide semiconductor fabrication.

Monolayers of TMDs exhibit unique photoluminescence (PL) properties due to their direct bandgap and strong excitonic effects. Quantum confinement in these materials leads to excitons (bound electron–hole pairs) with high binding energies, on the order of 0.5 eV, which is 10–100 times greater than in traditional inorganic and organic semiconductors.^5^ These 2D semiconductors hold immense potential for transformative optoelectronic applications in fields such as sensing, lighting, imaging, and information processing.^6,7^ A key to realizing their potential for novel optoelectronic devices lies in controlling their light emission and absorption properties.

One approach to achieving this control is through electrostatic doping, which alters the charge carrier concentrations in TMDs.^8^ Counter-doping the intrinsic excess of electrons or holes in the material effectively suppresses nonradiative recombination pathways, thereby enhancing quantum efficiency.^9^ Doping can be used to regulate the population of charged excitons and to introduce polaronic effects.^10^ Electrostatic doping has been successfully employed to enhance PL^9,11^ and second-harmonic generation^12^ in TMD monolayers, as well as to modulate the light-matter coupling strength between TMD excitons and optical cavities.^13−16^

The emission and absorption properties of 2D semiconductors can be substantially modified by integrating them with photonic structures. Coupling with optical cavities alters the local density of optical states in TMDs, enhancing light-matter interactions, modifying emission spectra, and boosting PL efficiency.^17,18^ Strong coupling has been demonstrated between TMD monolayers and various optical cavities,^16,19,20^ including microcavities,^21−23^ photonic crystal cavities,^24^ and plasmonic nanocavities.^25,26^ Previous studies have reported increased PL intensity in microcavities and photonic crystal cavities,^27^ as well as in plasmonic nanostructures relying on localized plasmon resonances.^20,28,29^ Moreover, lasing has been achieved with optically pumped TMDs coupled to photonic crystal cavities^30−33^ and planar microcavities.^34,35^

A periodic array of metal nanoparticles, where the interparticle distance matches the wavelength of diffracted light, presents a versatile approach that allows for simple fabrication and straightforward engineering of the lattice geometry and unit cell to control emission angles and polarization.^36−40^ These plasmonic lattices offer distinct advantages over traditional photonic structures by providing an open, spatially extended cavity that is more adaptable to the active material.^41^ Plasmonic lattices coupled with active materials, such as organic molecules and quantum dots, have been successfully utilized for strong light-matter coupling,^42−44^ as well as for lasing and Bose–Einstein condensation.^42,45−49^ Distinct characteristics of plasmonic lattices include the ability to generate polarization patterns^40,50^ and exhibit long-range spatial coherence.^51,52^ Aside from the pioneering works on strong coupling with 2D semiconductors^13,53−55^ and a few earlier studies involving polymer semiconductors^56^ and liquid crystals,^57^ previous research on plasmonic lattices has largely focused on active materials restricted to optical excitation.

In this work, we demonstrate control over the PL of 2D semiconductors integrated with plasmonic lattices. Our results show that plasmonic lattices can significantly modulate the PL intensity and directionality of TMD monolayers. Importantly, we introduce gate electrodes to actively tune the PL efficiency of the 2D semiconductors coupled to plasmonic lattices.

## Results and Discussion

**Figure 1:**
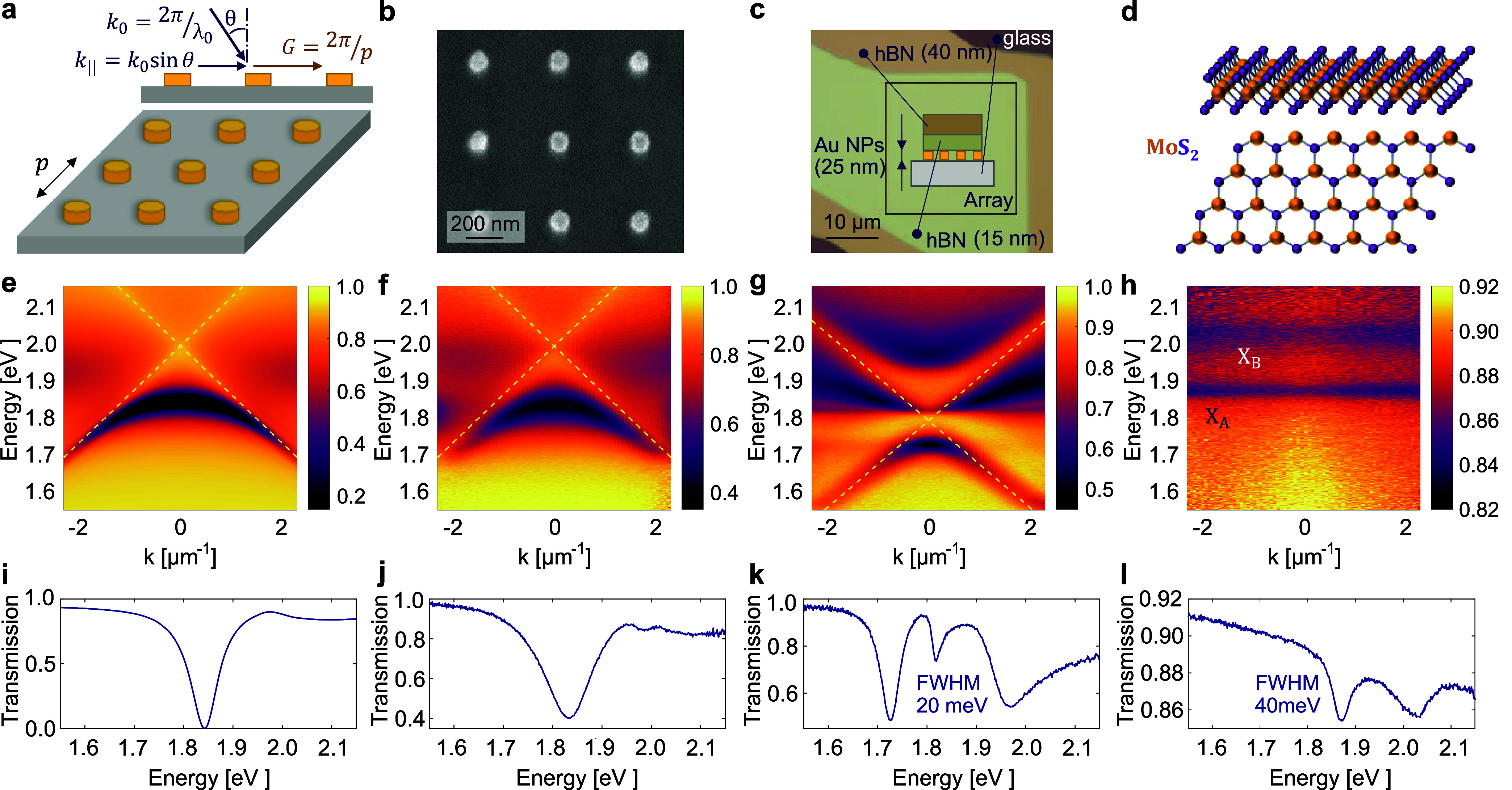
Dispersion of nanoparticle arrays and monolayer MoS_2_. (a) Illustration of a nanoparticle array. Here, λ_0_ and *k*_0_ are the free-space wavelength and wave vector of incident light, respectively. Incident angle is denoted as θ, and the in-plane scattered component of the wave vector is *k*_||_. Periodic lattice with period *p* causes a momentum kick *G* that adds to the in-plane momentum. (b) Scanning electron microscope image of a nanoparticle array. (c) Schematic of Au nanoparticle (NP) array covered with two flakes of hBN on a glass substrate. (d) Schematic of the atomic structure of monolayer molybdenum disulfide (MoS_2_). (e) Simulated transmission of a nanoparticle array in the coupled dipole approximation. White-light transmission measurements of (f) a bare array, (g) an array with two hBN flakes on top, and (h) a monolayer MoS_2_ sandwiched between two hBN flakes on a glass substrate. Yellow dashed lines in (e–g) show the light lines. In (h) *X*_*A*_ and *X*_*B*_ denote the *A* and *B* excitons, respectively. Crosscuts along *k* = 0 are shown in the bottom row (i–l).

A schematic of the nanoparticle array is shown in Figure 1a. A square array of Au nanoparticles is fabricated on a glass substrate by electron-beam lithography (EBL) and electron-beam evaporation. The periodic arrangement of nanoparticles gives rise to collective plasmonic-photonic modes called surface lattice resonances (SLRs) due to the strong coupling between the localized single-particle plasmon resonances and the diffractive orders of light.^36,38^ While the localized surface plasmons confine light into a subwavelength space around the nanoparticles, the long-range coupling via diffractive orders enables a collective response and higher quality (*Q*) factors. The *Q* factor of localized surface plasmon resonances is generally below 10, whereas the *Q* factor of SLR modes can reach several hundreds.^38^ A scanning electron microscope image of an array of cylindrical nanoparticles is shown in Figure 1b.

After fabricating the arrays, a stack of 2D materials is transferred on top of the array using a standard dry pick-up and transfer method.^58^Figure 1c shows the schematic of the part of the sample where two flakes of hBN overlap the array. The final stack consists of a monolayer TMD (molybdenum disulfide (MoS_2_), Figure 1d) sandwiched between two hBN flakes. The dielectric hBN encapsulation protects against oxidation, provides electrical insulation, and improves the optical response of TMD monolayers.^59^ Furthermore, the bottom hBN prevents quenching of the PL by the metallic nanostructures; quenching increases significantly below a distance of 10 nm.^60^ However, TMDs should be placed close to the nanoparticles to benefit from plasmonic enhancement that facilitates coupling. As shown by the numerical simulations of the electric field for the SLR modes in Supporting Information,Figure S1, the field rapidly decays orthogonally from the surface of the array. Therefore, we use a bottom hBN of thickness between 10 and 15 nm. For the PL measurement, a 633 nm laser is focused onto the sample with an objective. The same objective is used to collect the emitted light in an inverted microscope configuration and guided to either an avalanche photodiode (APD) or the entrance slit of a spectrometer and imaged with a charge-coupled device (CCD) camera. We optically characterize the samples by spatial PL maps and angle-resolved spectra. In the samples equipped with gate electrodes, we apply a bias voltage and measure the gate voltage dependent transmission and PL spectra. See Methods for details on sample fabrication and experiments.

Figure 1e presents the simulated and Figure 1f the measured dispersion relation of the transverse electric (TE) mode of the SLR excitations. The simulation is performed using coupled dipole approximation; see Supporting Information, Section S1, for a description of the model. We use a linear polarizer perpendicular to the spectrometer slit to select the TE mode (*E*_*x*_, *k*_*y*_) for analysis. The dispersion of the transverse magnetic (TM) mode (*E*_*y*_, *k*_*y*_) can be found in Supporting Information, Figure S2. As depicted in Figure 1g, the application of the 2D materials results in a significant red shift of the SLR dispersion, attributed to the high refractive index of around 2 of the hBN.^61^ Due to the red shift, we observe the upper SLR dispersion band with a peak around 1.97 eV in Figure 1k. Additionally, the introduction of the dielectric hBN layer leads to the outcoupling of the quadrupolar SLR mode at *k* = 0, manifested as a narrow peak at 1.82 eV in Figure 1k. While in an infinite periodic array with a symmetric refractive index environment, the quadrupolar mode does not radiate to the far field at *k* = 0, in a finite system with defects, such as sample edges or a surrounding higher-refractive index material, the quadrupolar mode can couple out.^45,49^ By fitting the SLR mode dispersions to the measured transmission spectra, we obtain the effective refractive index, which shifts from 1.52 for the glass substrate to 1.69 due to the added hBN. See Supporting Information, Section S3, for a description of the SLR dispersion relations. The transmission of a monolayer MoS_2_ sandwiched between two hBN flakes, in Figure 1h,l, shows that the absorption peaks for MoS_2_*A* and *B* excitons occur around 1.87 and 2.03 eV, respectively.

**Figure 2:**
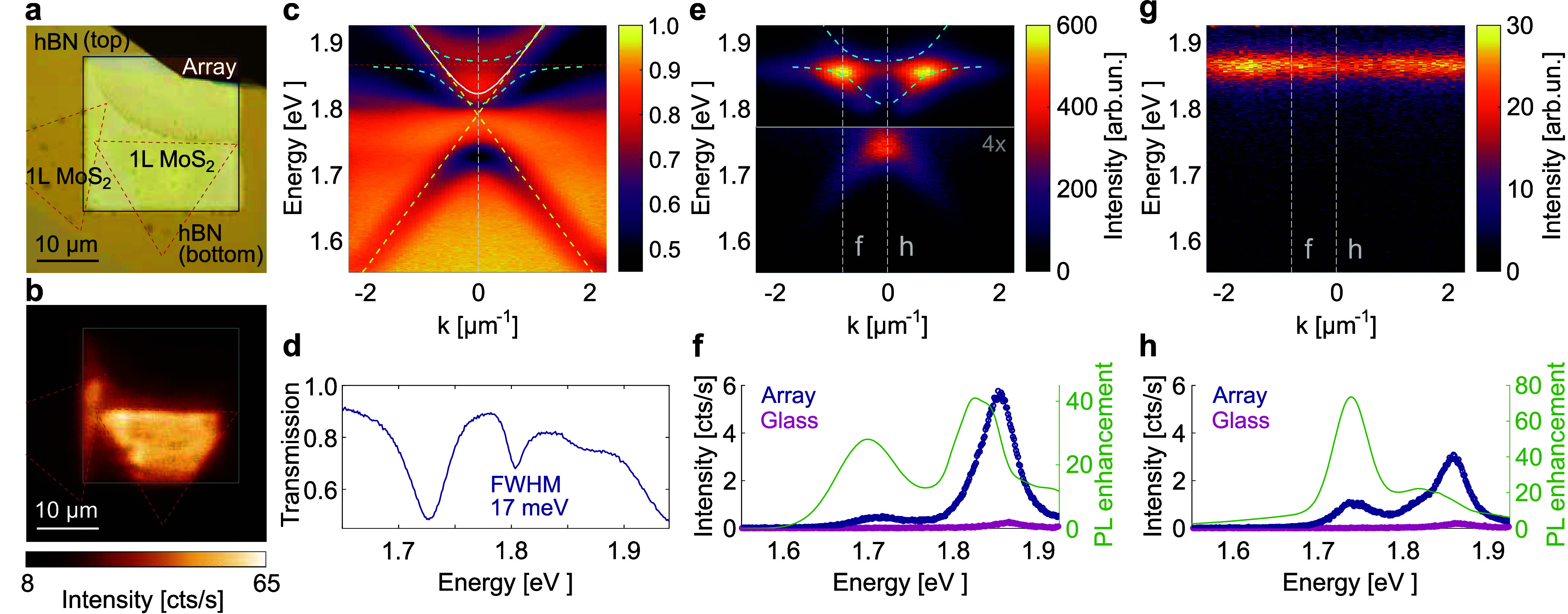
Photoluminescence (PL) enhancement of a monolayer MoS_2_ coupled to a nanoparticle array. (a) Microscope image of the sample consisting of a nanoparticle array and MoS_2_ monolayers, which partially overlap with the array and are sandwiched between hBN flakes. (b) Spatial PL map of the sample. (c) Angle-resolved white-light transmission spectrum of the MoS_2_ on the array and (d) crosscut along *k* = 0. Yellow dashed lines in (c) show the light lines and cyan dashed curves indicate the upper and lower polariton bands obtained from the coupled modes fitting. Angle-resolved PL spectra of the MoS_2_ monolayer (e) on array and (g) on glass. Bottom part of (e) is multiplied by 4 for better visibility of the features. Energies of the upper and lower polariton bands are indicated by cyan dashed lines, and the exciton energy by horizontal red dashed line. Figures (f,h) show the crosscuts along the vertical white dashed lines in (e,g) and the corresponding PL enhancement factors.

Measuring the sample transmission in the overlap region of the MoS_2_ flake and the array, as shown in Figure 2c, we observe an additional splitting of the upper SLR dispersion. The lower part of the SLR dispersion remains unaltered, as it is far from the exciton. By fitting the coupled oscillator model^44^ to the measured dispersions, we obtain a Rabi splitting Ω_R_ = 52 meV; see Supporting Information, Section S3, for details of the model and fitting. This is larger than the line widths of the SLR mode (γ_SLR_ = 20 meV) and the *A* exciton (γ_X_ = 40 meV), and fulfills the general criterion for strong coupling, Ω_R_ > (γ_SLR_ + γ_X_)/2.^13,54,55,62,63^

Next, we compare the PL properties of the hBN-encapsulated TMDs positioned on top of the nanoparticle array versus on a glass substrate. A microscope image of the sample is shown in Figure 2a. The spatial PL map in Figure 2b clearly demonstrates that the PL is enhanced in the regions where the TMD monolayers are overlapped with the array. The enhancement arises from the Purcell effect, where plasmonic nanoparticles modify the local density of optical states, thereby increasing the radiative decay rate of emitters. Emitters located near the array excite the SLRs, which scatter light in directions determined by their dispersion relation. Angle-resolved emission spectra in Figure 2e,g reveal that the highest enhancement occurs where the PL emission line of the TMD monolayer intersects with the lower polariton band. The crosscuts in Figure 2f,h show that the PL is enhanced by a factor of 40–80 when the TMD is on the array compared to on glass. While the absolute PL intensity is highest at off-normal angles, the relative enhancement is greatest at the dispersion band edge, which is far from the PL peak of the TMD. We also investigated the sample under pulsed excitation and found that when pumping with a femtosecond-pulsed laser, the enhancement factors can be even higher, 2 orders of magnitude (see Supporting Information, Section S4 and Figure S3).

In a square array, the SLR dispersion band edge at *k* = 0 occurs around λ = *pn*_eff_, where *p* is the lattice period and *n*_eff_ is the effective refractive index. Therefore, the SRL dispersion bands can be shifted in energy by changing the lattice periodicity.^36−38^ The flexibility to tune the geometry of the array allows for control over the direction of the PL emission. To illustrate this, we fabricated arrays with the band edge (at *k* = 0) tuned closer to the PL spectrum of the TMD. As presented in Supporting Information, Figure S4, the enhanced PL is emitted at *k* = 0 (i.e., perpendicular to the surface) in this configuration. Previous studies have shown that both the array geometry^64^ and the refractive index^65^ can be dynamically varied, offering further opportunities for precise control over the out-coupled emission.

**Figure 3:**
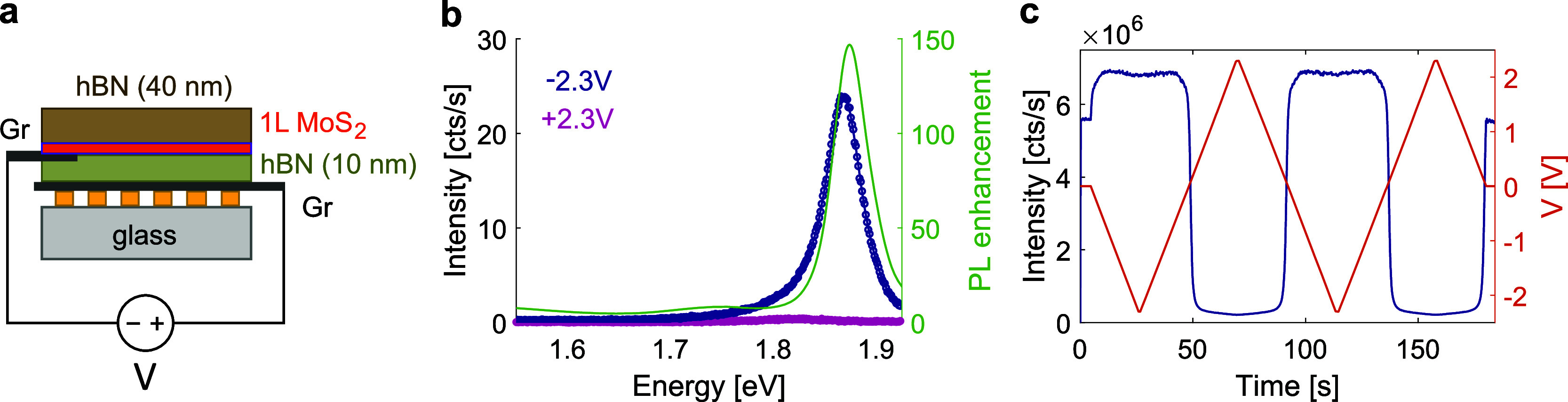
Gate-controlled photoluminescence (PL) of a monolayer MoS_2_ coupled to a nanoparticle array. (a) Schematic of the sample. (b) PL spectra from the MoS_2_ on array at −2.3 V versus +2.3 V applied bias and the corresponding PL enhancement factor. (c) Time trace of PL intensity recorded with APD (i.e., integrated over all collection angles and wavelengths) while sweeping the bias voltage between −2.3 and +2.3 V.

Now we turn to the electrical tuning of the PL under applied bias voltage. We apply gate electrodes to the sample to regulate the charge carrier concentration in the TMD monolayer, thereby altering the PL efficiency. A schematic of the sample is shown in Figure 3a; a microscope image can be found in Supporting Information, Figure S5. One (multilayer) graphene (Gr) flake is placed at the bottom of the stack and connected to a prepatterned Au electrode. Another Gr flake connects the TMD to a second Au electrode, and voltage is applied between the two two Gr flakes. For this device, we used an array of rod-shaped nanoparticles; see Methods for details of the sample geometry. As with the sample without electrodes, the Rabi splitting, Ω_R_ = 46 meV, extracted from the coupled oscillator model fulfills the strong coupling criterion, Ω_R_ > (γ_SLR_ + γ_X_)/2, where γ_SLR_ = 40 meV and γ_X_ = 40 meV. In Supporting Information, Figure S5, we present the angle-resolved white-light transmission and PL spectrum of the sample as well as spatial PL maps obtained for different bias voltages. As shown in Figure 3b, the PL intensity can be significantly modulated—either nearly fully suppressed or enhanced by more than 2 orders of magnitude—through the application of bias voltage. Remarkably, due to the thin (10 nm) hBN gate dielectric, the required bias voltage to achieve this control is below 2 V. A thinner gate dielectric provides a higher capacitance *C* = ϵ_0_ϵ_r_*A*/*d*, where *A* is the area and *d* the thickness. The higher capacitance means that for the same applied voltage, more charge is induced in the TMD layer (*Q* = *CV*_gate_). This results in a bias voltage ten times lower than in studies employing thick SiO_2_ gate dielectrics to electrostatically dope TMDs for PL control,^9,11^ underscoring the efficiency of our approach.

From the time trace of PL as a function of applied voltage in Figure 3c, we can see that the maximum PL intensity is reached at around −0.7 V. Previous studies have indicated that the PL efficiency of electrostatically doped TMD monolayers is highest at charge neutrality.^9,11^ A negative applied bias in our configuration means drawing electrons out from the TMD, so the observation of maximum PL efficiency at the negative bias side is consistent with the fact that MoS_2_ is usually intrinsically n-doped. On the positive bias side, the TMD moves away from charge neutrality as it becomes overcrowded with electrons. In this case, nonradiative recombination takes over, mostly due to charged excitons,^9^ and consequently, the PL from neutral excitons is suppressed. For the same reason, applying a higher negative bias than −0.7 V does not lead to further enhanced PL; the TMD becomes depleted of electrons (or filled with holes). However, as hole injection into MoS_2_ is not efficient,^66^ the PL efficiency does not drop quickly upon applying higher negative bias.

**Figure 4:**
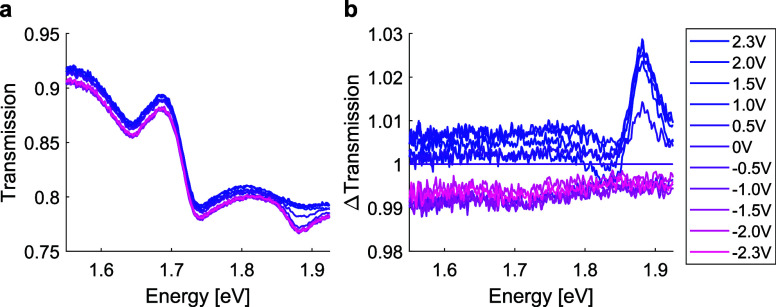
Transmission as a function of applied voltage. (a) Transmission spectrum of the MoS_2_ on array equipped with Gr electrodes, as a function of applied voltage. (b) Transmission spectra in (a) normalized to the 0 V spectrum.

To further investigate the origin of the observed changes in PL intensity, we measured the transmission of the sample as a function of applied voltage. The transmission measurements in Figure 4a,b show that the sample becomes only slightly more transparent (around 3%) close to the exciton energy when applying a positive bias. Therefore, we exclude bias voltage-related changes in the sample absorption or reflectance as the cause of the observed PL enhancement, but attribute the effect mostly to changes in the recombination pathways. As shown in Figure 4a, under a negative bias (from −2.3 to 0 V), a dip in transmission appears around 1.87 eV, corresponding to absorption by the neutral exciton. Upon applying a positive bias, this dip becomes shallower, indicating a reduction in neutral exciton absorption. The transmission normalized to the 0 V spectrum in Figure 4b reveals that while neutral exciton absorption decreases, charged exciton (trion) absorption increases, with a new dip appearing around 1.83 eV. This supports our interpretation of the PL intensity modulation described above. Additionally, we observe an overall baseline shift in transmission when transitioning from negative to positive bias, which we attribute to changes in the absorption of graphene due to doping.^67^

## Conclusions

In summary, we have demonstrated control over the PL properties of TMDs by coupling them to periodic plasmonic nanostructures and tuning their charge carrier concentration through gate electrodes. Our results show that both the PL intensity and emission angle of TMD monolayers can be controlled by varying the geometry of the nanoparticle array. This control arises from the coupling between the SLRs of the plasmonic lattice and the excitons in the TMDs, resulting in significant changes to the emission spectrum and angular distribution of the PL. Additionally, we have shown that the PL intensity of TMDs can be effectively controlled via electrostatic doping. By applying a low bias voltage of around 2 V we changed the PL intensity by over 2 orders of magnitude.

Our work highlights the potential of plasmonic lattices to modify the emission properties of 2D semiconductors. Electrical control introduces new ways to tune light emission that are not available with common active materials, like organic molecules and quantum dots. This represents a significant advancement toward the development of electrically modulated and powered nanoscale light sources based on plasmonic lattices. Besides integrated light sources, the ability to control the emission and absorption of TMDs electrically can be applied to photodetectors and optical modulators. Given the short lifetimes of the plasmonic modes, the plasmon-enhanced coupling can lead to faster response times in the subpicosecond (THz frequency) range.^47,48^ Additionally, TMDs show potential for quantum information processing as single-photon emitters,^68^ and plasmonic lattices enable outcoupling of single-photon emission without the need of precisely positioning the emitter within the cavity.^41^ Electrically tunable emission is also useful for chemical and biological sensing applications, where plasmonic lattices can provide high sensitivity and direct access to analytes.^69^

In addition to advancing techniques for electrical control and excitation, our findings open new avenues for fundamental research on 2D semiconductors coupled to plasmonic structures, including the potential to control the electronic spin of 2D semiconductors through the valley degree of freedom using chiral plasmonic nanostructures.^70^ Engineering lattice geometry further enables the exploitation of nontrivial optical effects, such as beam steering^71^ and topological invariants.^72−75^ Combining lattice geometry engineering with electrical control of 2D semiconductors offers a versatile platform for fundamental studies and a pathway toward novel nanoscale optoelectronic devices with customized emission characteristics.

## Methods

### Sample Fabrication

Square arrays of Au nanoparticles were fabricated on a borosilicate glass substrate using EBL and electron-beam evaporation. Prior to EBL, the glass substrates were treated with O_2_ plasma and spin-coated with a 200 nm thick layer of 950 K poly(methyl methacrylate) (PMMA) resist. After patterning the arrays, the PMMA was developed in a 1:3 ratio of deionized water to isopropanol solution. A 3 nm Ti adhesion layer and a 25 nm Au layer were then evaporated onto the patterned PMMA, followed by lift-off in acetone. Nanoparticles in the sample with hBN-MoS_2_-hBN (Figures 1 and 2) were cylindrical, with a diameter of 100 nm and a height of 25 nm. The array period was 410 nm, and the array size was 25 × 25 μm^2^. In the sample with electrodes (Figures 3 and 4), the nanoparticles were rod-shaped with a width of 80 nm, a length of 276 nm (corresponds to 65% of the period), and a height of 25 nm. The array period was 425 nm, and the array size was 25 × 25 μm^2^. We chose Au as the nanoparticle material because it is chemically inert and does not oxidize; other metals, such as Ag or Al, could also be used.^36,38^

The stack of van der Waals materials was constructed using a polymer-based dry pick-up and transfer method.^58^ First, Gr (NGS Naturgraphit GmbH, Germany) and hBN (National Institute for Materials Science, Japan) flakes were mechanically exfoliated onto SiO_2_/Si substrates. The flakes with the desired thicknesses were initially selected by color contrast under an optical microscope, and their thickness was confirmed by scanning with an atomic force microscope. The chemical vapor deposition-grown monolayer MoS_2_ flakes (2D Semiconductors) were picked up directly from SiO_2_/Si substrates. The stack of 2D materials was assembled in a glovebox filled with Ar gas by using a polydimethylsiloxane (PDMS) stamp covered with a polycarbonate (PC) film. As a first step, the top hBN flake (40–50 nm) was picked up by contacting it with the PC-covered stamp at 80 °C. In the following steps, this hBN was used to sequentially pick up a TMD monolayer and a bottom hBN (around 15 nm), or, in the case of gated samples, a top contact Gr, a dielectric barrier hBN (below 10 nm), and a bottom contact Gr at 70–80 °C. The hBN encapsulation protects the TMD monolayers against oxidation and prevents quenching of the PL by the metallic nanostructures. The stack was then transferred on top of a nanoparticle array on a glass substrate by melting the PC film at a temperature above 175 °C. In the gated samples, the glass substrates were prepatterned with metal electrodes by photolithography and electron-beam evaporation of Ti/Au(5/50 nm). After transfer, the residual PC film was dissolved in chloroform. For refractive index matching, the samples were covered with either immersion oil and a cover glass (for samples without electrodes) or a PMMA layer (for samples with electrodes). Electrical connections between the sample and the voltage source were established by wire bonding.

### Optical Characterization

For optical characterization, the samples were mounted on a Nikon TE300 inverted microscope and measured using a custom-built experimental setup under ambient conditions. For PL measurements, the samples were excited with a continuous-wave He–Ne laser at 633 nm, focused onto the sample through the same objective (Nikon, 100×, 0.9 numerical aperture (NA)) used for detection (see Supporting Information, Figure S6 for the setup schematic). To block the reflected pump laser, we used a long-pass filter with a cutoff wavelength of 633 nm in the detection path. Angle-resolved measurements were performed by collecting the light emitted or transmitted by the sample, with the back focal plane of the objective focused onto the entrance slit of a spectrometer (Princeton Instruments Acton SpectraPro 300i) equipped with a CCD camera (Princeton Instruments BLAZE 400).

The angle of light scattered by the sample, denoted as θ_*y*_, is mapped onto the spectrometer slit as *k*_*y*_ = *k*_0_ sin θ_*y*_ = 2 π/λ_0_ sin θ_*y*_, where λ_0_ is the free-space wavelength of the light. Each vertical position on the slit, and thus each pixel row on the CCD camera array, corresponds to a *k*_*y*_ value, while the pixel columns of the CCD camera represent the dispersed wavelengths, such that *E* = *hc*/λ = *hc*/(2π/*k*_*y*_) where *h* is Planck’s constant and *c* is the speed of light. Dispersion relations were obtained through white-light transmission measurements, where light from a broadband halogen lamp was focused onto the sample by a second objective (Olympus, 10×, 0.3 NA) from the top side, and the angle-resolved spectrum of the transmitted light was recorded with the spectrometer and CCD camera. Polarization was analyzed by placing a linear polarizer in the detection path. An iris was also placed in the detection path to spatially restrict the light collection area. Spatial PL maps were acquired by moving the sample with a nanopositioning stage and collecting the emitted light at each position using an APD (Excelitas SPCM-AQRH). For the time trace in Figure 3g, the APD count rates were corrected for the nonlinear response at high count rates due to the sensor’s dead time (24 ns). However, no correction was applied to the spatial PL maps, as the count rates are lower and the APD response is approximately linear below ∼1 MHz rates. Electrostatic doping of the samples was realized by connecting the sample to a direct-current voltage source (Keithley Instruments 2602B) and varying the applied gate voltage.

For femtosecond-pulsed laser excitation (Supporting Information, Section S4 and Figure S3), the sample was excited using a Ti-sapphire laser (Coherent Mira, 80 MHz, 800 nm, 200 fs), which drives an optical parametric oscillator (Coherent Mira OPO) to convert the output wavelength to 600 nm. The sample was mounted on a Nikon TE300 inverted microscope, and the light was collected in an analogous setup as the one described above using an objective with the same specifications (Nikon, 100×, 0.9 NA). The angle-resolved PL spectrum was analyzed using a spectrometer (Acton SP-2300) equipped with a CCD camera (Princeton Instruments PIXIS 100). A long-pass filter with a cutoff wavelength of 633 nm, a linear polarizer, and an iris to restrict the light collection area were used in the detection path.

## Supporting Information Available

Electric field for the surface lattice resonance at *k* = 0; dispersion relation of transverse magnetic (TM) SLR mode; photoluminescence (PL) spectra using femtosecond-pulsed laser excitation; photoluminescence (PL) enhancement with the SLR band edge tuned to a higher energy; gate-controlled photoluminescence (PL); additional data; schematic of the experimental setup; coupled dipole approximation; numerical simulations of the electric field; coupled oscillator model fits; and pulsed laser excitation (PDF)
