# Supplementary material for: Electrical Control of Photoluminescence in 2D Semiconductors Coupled to Plasmonic Lattices
Source: ACS Nano. 2025 Jan 20;19(4):4731–8. doi: 10.1021/acsnano.4c15459 (PMC11803915; doi:10.1021/acsnano.4c15459)
Supplement: Supplementary file 1 — nn4c15459_si_001.pdf [file nn4c15459_si_001.pdf]

Supporting Information: Electrical control of photoluminescence in 2D semiconductors coupled to plasmonic lattices

Antti J. Moilanen,*,† Moritz Cavigelli,† Takashi Taniguchi,‡ Kenji Watanabe,¶ and Lukas Novotny†

†*Photonics Laboratory, ETH Zürich, CH-8093 Zürich, Switzerland*

‡*Research Center for Materials Nanoarchitectonics, National Institute for Materials Science, 1-1 Namiki, Tsukuba 305-0044, Japan*

¶*Research Center for Electronic and Optical Materials, National Institute for Materials Science, 1-1 Namiki, Tsukuba 305-0044, Japan*

E-mail: amoilanen@ethz.ch

List of contents:

Figure S1. Electric field for the surface lattice resonance at $k = 0$.

Figure S2. Dispersion relation of transverse magnetic (TM) SLR mode.

Figure S3. Photoluminescence (PL) spectra using femtosecond-pulsed laser excitation.

Figure S4. Photoluminescence (PL) enhancement with the SLR band edge tuned to a higher energy.

Figure S5. Gate-controlled photoluminescence (PL); additional data.

Figure S6. Schematic of the experimental setup.

Section S1. Coupled dipole approximation

Section S2. Numerical simulations of the electric field

Section S3. Coupled oscillator model fits

Section S4. Pulsed laser excitation

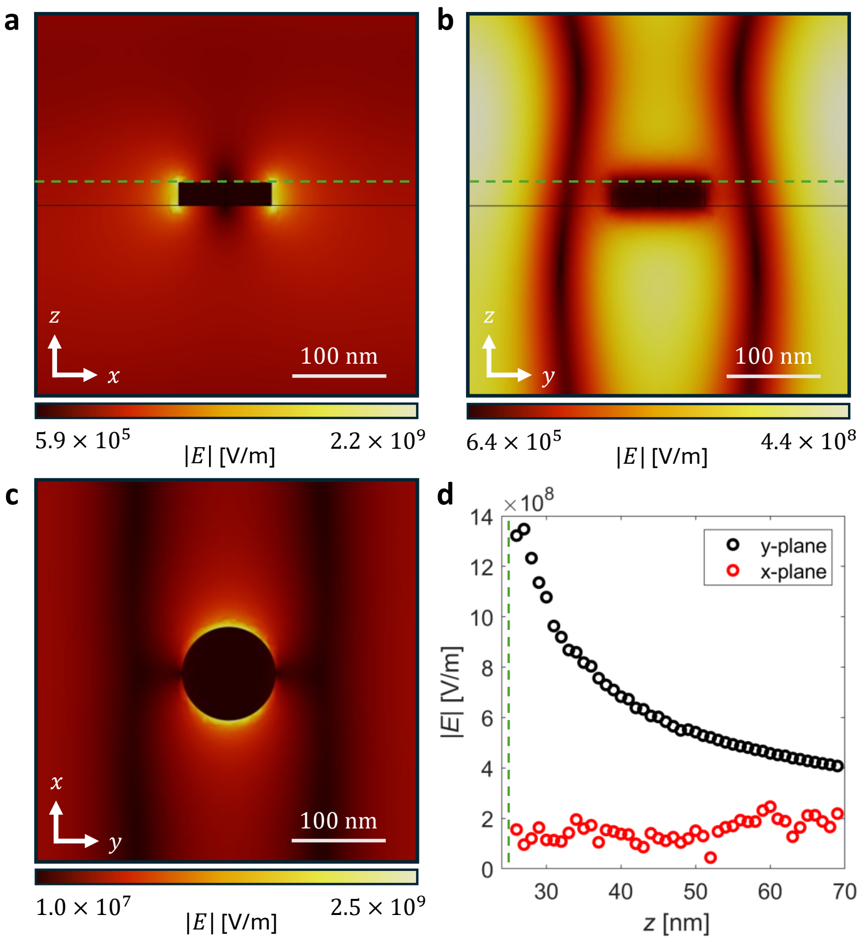

Figure S1: Electric field for the surface lattice resonance at $k = 0$. The simulation assumes periodic boundary conditions in the x- and y-directions, with a period of 415 nm. The Au nanoparticle has a height of 25 nm and a diameter of 100 nm. The refractive indices of both the substrate and superstrate are 1.52, and the incident electric field is x-polarized. (a) Cross-section along the y-plane ($y = 0$). (b) Cross-section along the x-plane ($x = 0$). (c) Crosscut along the z-plane at the nanoparticle surface ($z = 25$ nm), indicated by the green dashed line in (a) and (b). (d) Electric field averaged over the unit cell as a function of z for both the y- and x-planes. The nanoparticle surface at $z = 25$ nm is marked by the green dashed line. In panels (a-c), the plots span 415 nm $\times$ 415 nm. See Section S2 for further description of the model.

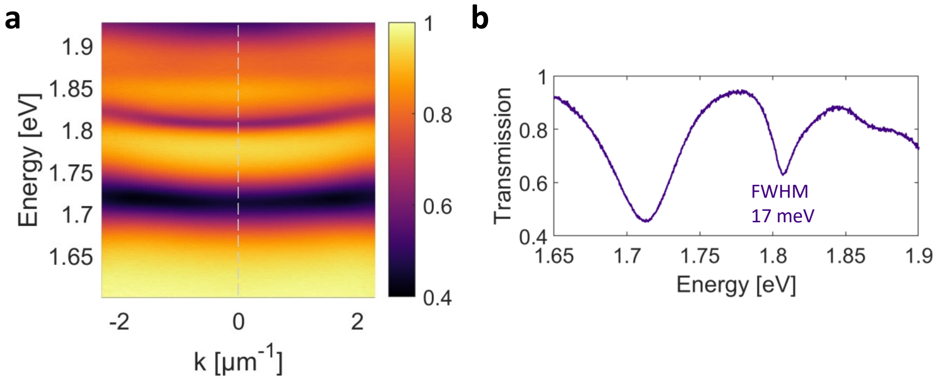

Figure S2: Dispersion relation of transverse magnetic (TM) SLR mode. (a) White-light transmission measurement of the sample with hBN-MoS_2-hBN on array, using a vertical polarizer in the detection. Within the collection angles, the parabolic SLR dispersion branches remain below the energy of MoS_2 absorption. (b) Crosscut along $k = 0$ with the linewidth of the upper SLR mode indicated.

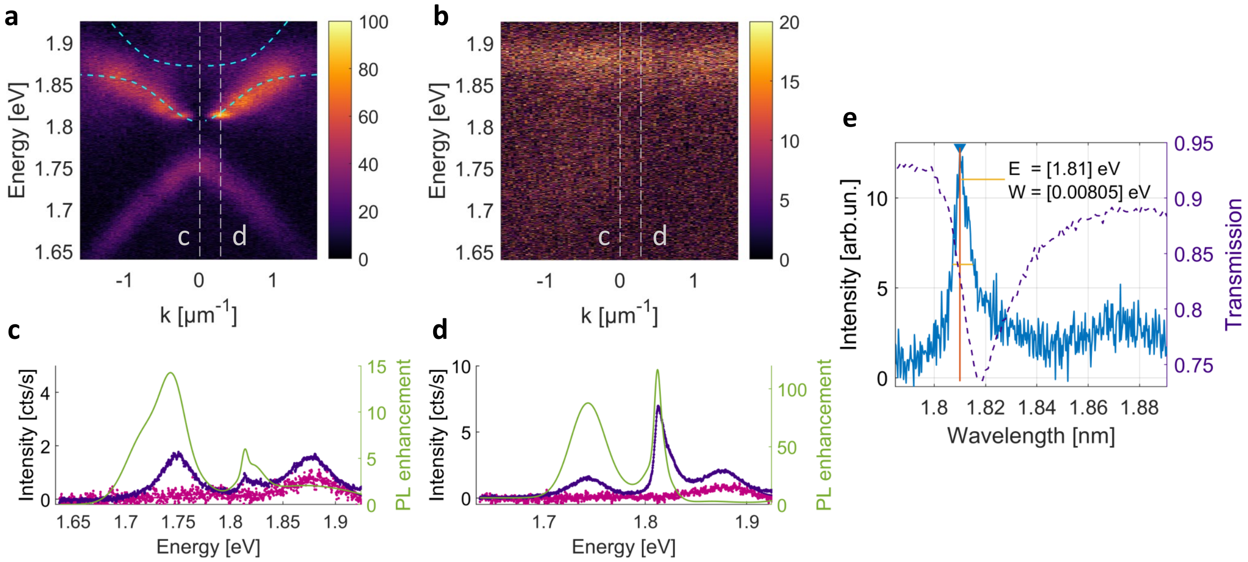

Figure S3: Photoluminescence (PL) spectra using femtosecond-pulsed laser excitation. The sample is the same as shown in the main text Figs. 1 and Fig. 2. Angle-resolved PL spectra of the MoS_2 monolayer (a) on array and (b) on glass. The energies of uncoupled SLR as well as upper and lower polariton bands are indicated by cyan dashed lines, as in main text Fig. 2e. Figures (c,d) shows the crosscuts along the white dashed lines in (a,b) and the PL enhancement factor. (e) Linewidth measurement of the crosscut (d) in Fig. 1. Purple dashed line is a crosscut at $k = 0$ from the transmission measurement shown in main text, Fig. 2c-d.

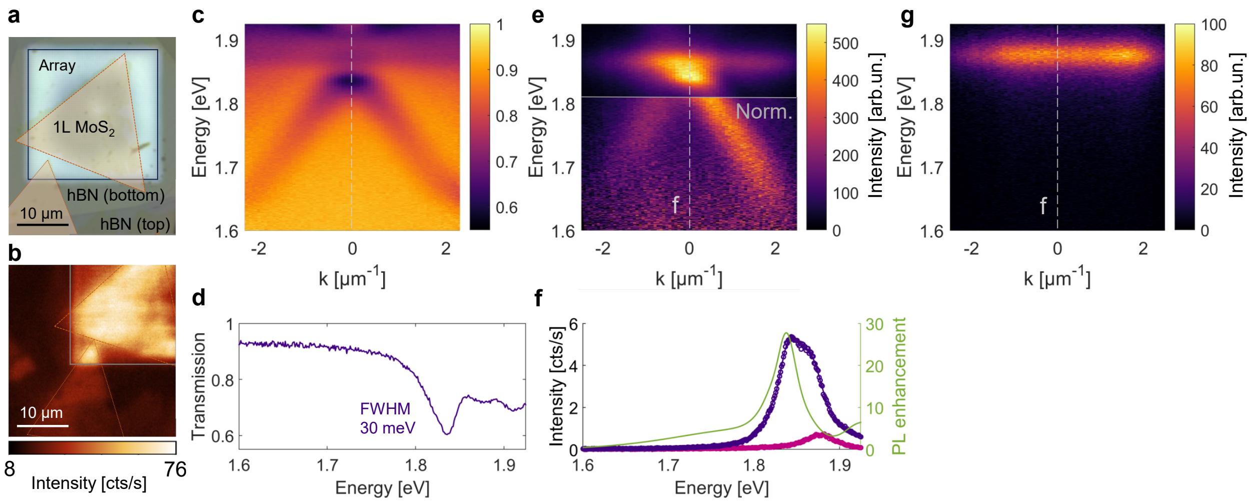

Figure S4: Photoluminescence (PL) enhancement with the SLR band edge tuned to a higher energy. (a) A microscope image of the sample consisting of a nanoparticle array and MoS_2 monolayers, which partially overlap with the array and are sandwiched between hBN flakes. (b) A spatial PL map of the sample. (c) Angle-resolved white-light transmission spectrum of the MoS_2 on the array and (d) crosscut along $k = 0$. Angle-resolved PL spectra of the MoS_2 monolayer (e) on array and (g) on glass. The bottom part of (e) is line-normalized for better visibility of the features. Figure (f) show the crosscuts along the vertical white dashed lines in (e, g) and the corresponding PL enhancement factor. The period of the nanoparticle array was 400 nm, and the cylindrical particles had a diameter of 90 nm and a height of 25 nm.

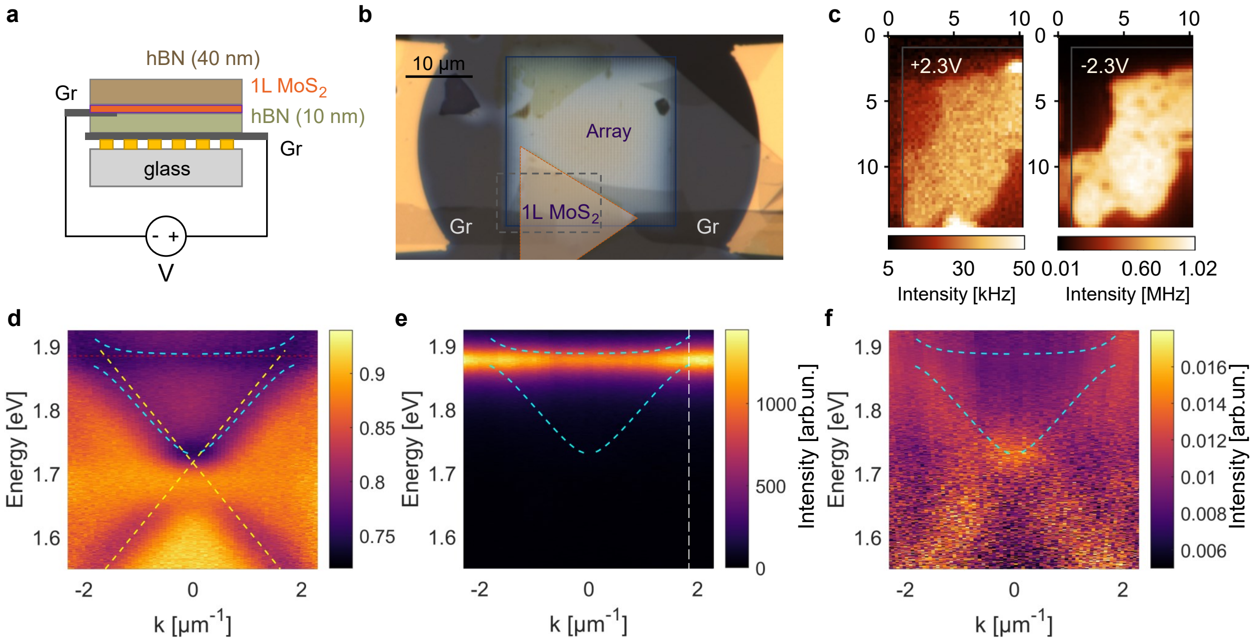

Figure S5: Gate-controlled photoluminescence (PL); additional data. (a) Schematic and (b) microscope image of the sample; a monolayer MoS_2 on a nanoparticle array equipped with Gr electrodes. (c) Spatial PL maps at $+2.3$ V (left) and -2.3 V (right) applied bias, obtained from the area marked by dashed rectangle in (b). (d) White-light transmission measurement of the MoS_2 on array equipped with Gr electrodes. Yellow dashed lines in (d) show the light lines, and cyan dashed curves indicate the upper and lower polariton bands obtained from fitting the coupled modes model. Compared to the sample without electrodes, the SLR mode in this sample has a larger linewidth of 40 meV; the increased linewidth can be attributed to larger nanoparticles and Gr absorption. (e) Angle-resolved PL spectrum of the MoS_2 on array at 0 V applied voltage. The PL spectra shown in the main text Fig. 3b are crosscuts along the vertical dashed line indicated in (e), at -2.3 V versus $+2.3$ V applied bias. (f) Angle-resolved PL spectrum of the MoS_2 on array at 0 V applied voltage; line-normalized for better visibility of the dispersion features.

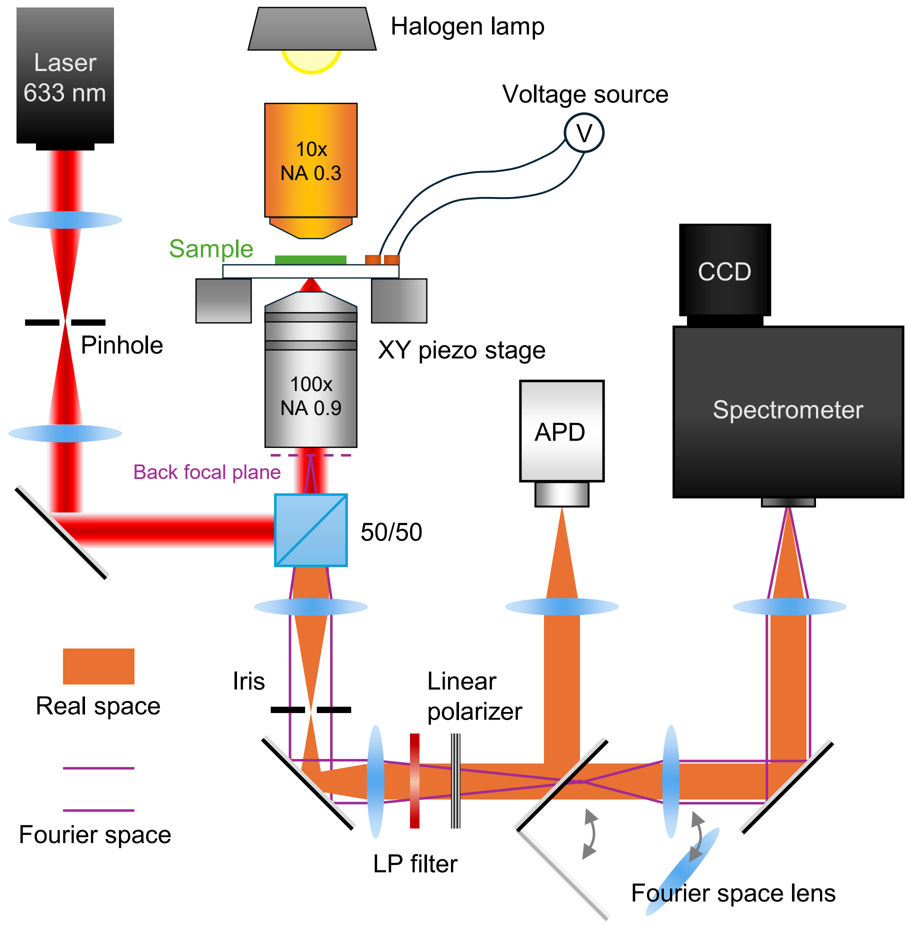

Figure S6: Schematic of the experimental setup. The sample is excited using a 633 nm continuous-wave laser through a 100× objective (NA 0.9). The same objective collects the sample luminescence, which is directed either to an APD or a spectrometer with a CCD camera for analysis. For angle-resolved (Fourier space) spectra, the back focal plane of the objective is focused on the spectrometer slit. For white-light transmission measurements, the sample is illuminated by a halogen lamp through a 10× objective (NA 0.3), and the transmitted light is collected by the 100× objective. For spatial photoluminescence maps, the sample position is scanned using a XY piezo stage, and the light from each position is collected by the APD. In gate-controlled experiments, the sample electrodes are connected to a voltage source. Further details on the measurements are provided in the Methods section of the manuscript.

Section S1. Coupled dipole approximation

Extinction spectrum of a periodic array of nanoparticles is calculated using the coupled dipole approximation (CDA).[1–3] In the model each nanoparticle is treated as a single electric dipole char-

acterized by its polarization

$$\vec{P}_n = \alpha(\vec{E}_n^{\text{inc}} + \vec{E}_n^{\text{sc}}), \tag{1}$$

where $\vec{E}_n^{\text{inc}}$ is the incident field acting on the nth particle, and $\vec{E}_n^{\text{sc}}$ is the field resulting from scattering by all the other nanoparticles. We calculate the polarizability α for an ellipsoid nanoparticle using the modified long-wavelength approximation.[3,4] The scattered field is expressed as

$$\vec{E}_n^{\text{sc}} = \sum_{m=1, m\neq n}^{N} \underline{\vec{G}}\left(\vec{r}_n, \vec{r}_m\right) \vec{P}_m, \tag{2}$$

where $\underline{\vec{G}}$ is the dyadic Green's function ($R = |\vec{R}| = |\vec{r}_n - \vec{r}_m|$) of the electric-field

$$\underline{\vec{G}}\left(\vec{r}, \vec{r}'\right) = \frac{e^{ikR}}{4\pi\epsilon_0 R}\left[\left(\underline{\vec{I}} - \hat{\vec{R}}\hat{\vec{R}}\right) + \frac{ikR-1}{k^2R^2}\left(\underline{\vec{I}} - 3\hat{\vec{R}}\hat{\vec{R}}\right)\right]. \tag{3}$$

The diagonal terms of the Green's function representing the self-interaction of the nanoparticle are excluded to avoid singularity.[2] Applying the scattered field to Eq. (1) leads to a set of linear equations

$$\sum_{m=1}^{N} \underline{\vec{A}}_{nm} \vec{P}_m = \vec{E}_n^{\text{inc}}, \tag{4}$$

where the interaction matrix $\underline{\vec{A}}$ elements are given by

$$\underline{\vec{A}}_{nm} = \begin{cases} \underline{\vec{\alpha}}^{-1}\underline{\vec{I}}, & n = m, \\ -\underline{\vec{G}}\left(\vec{r}_n, \vec{r}_m\right), & n \neq m. \end{cases} \tag{5}$$

The interaction matrix $\underline{\vec{A}}$ contains the single nanoparticle polarizabilities in the diagonal, representing the self-interaction of the nanoparticles. Solving the induced polarization $\vec{P}_m$ of the nanoparticles from Eq. (4), we can calculate the the extinction cross section:[1]

$$C_{\text{ext}} = \frac{4\pi k}{|E^{\text{inc}}|^2} \sum_{n=1}^{N} \text{Im}\left(\vec{E}_n^{\text{inc}*} \cdot \vec{P}_n\right). \tag{6}$$

The results shown in main text Fig. 1 (we approximate transmission equal to $1-$extinction) were calculated for 30×30 array of nanoparticles excited with a plane wave polarized in the x-direction. The parameters were adopted directly from the experiments: particle diameter was set to 100 nm, height 25 nm, and period 410 nm. Background refractive index was set to 1.52 and the permittivity of gold was obtained from tabulated values.[5]

Section S2. Numerical simulations of the electric field

Numerical simulations are conducted using COMSOL Multiphysics® 6.2 with the Wave Optics module. The optical response of the nanoparticle array is modelled by a unit cell containing a single nanoparticle, with Floquet-periodic boundary conditions applied at the lateral (x and y) boundaries and perfectly matched layers (PML) implemented in the vertical (z) direction. The refractive indices of both the substrate and superstrate are 1.52, and the optical constants for crystalline gold are obtained from Johnson and Christy.[5] To assess the near-field enhancement effects associated with the surface lattice resonances, the system is excited by a plane wave at zero incident angle. In the results shown in Fig. S1, the nanoparticle is cylindrical with a diameter of 100 nm and a height of 25 nm, and the period in x and y is 415 nm. The incident electric field is x-polarized.

Section S3. Coupled oscillator model fits

The dispersion relations of SLR modes in the empty lattice model are given by[6,7]

$$E_{\text{SLR}}(k_x, k_y) = \frac{\hbar c}{n_{\text{eff}}} \sqrt{\left(\frac{2n\pi}{p} + k_x\right)^2 + \left(\frac{2m\pi}{p} + k_y\right)^2}, \tag{7}$$

where $\hbar$ is reduced Planck's constant, c is the speed of light, n_{eff} is the effective refractive index within the mode volume, p is the lattice period, and (n, m) denote the diffractive orders. Here we restrict to square lattices with equal periodicity in x and y. Equation (7) yields linear dispersions that cross at the Γ-point of the lattice. Finite size of the nanoparticles leads to scattering that

couples the counter-propagating modes to each other, resulting to an opening of a band gap in the dispersion at $k = 0$. We fit the dispersion lines given by Eq. (7) to the measured dispersions to obtain n_{eff}, excluding from the fits the data points around Γ-point to account for the band bending close to the band gap. Solving the eigenvalues of the coupled modes Hamiltonian,[8,9]

$$H = \begin{pmatrix} E_{\text{SLR}} - i\gamma_{\text{SLR}} + s & g \\ g & E_{\text{X}} - i\gamma_{\text{X}} \end{pmatrix}, \tag{8}$$

yields the energies of the upper and lower polariton branches. Here, γ_{SLR} is the SLR mode linewidth and E_{X} and γ_{X} are the exciton (absorption) energy and linewidth, respectively. We note that while some of the previous works use as γ the FWHM of the modes,[9–11] others use the half-width at half maximum (HWHM).[12–14] In this work, we used the FWHM, extracted by Lorentzian fits to the peaks in the extinction spectra, as the linewidths $\gamma_{\text{SLR,X}}$.

The coupling strength g corresponds to the Rabi splitting $\Omega_{\text{R}} = \sqrt{4g^2 - (\gamma_{\text{SLR}} - \gamma_{\text{X}})^2/4}$ observed in the transmission measurements of the coupled system. We follow the procedure in Ref.[9] and include a parameter s in Eq. (8) to take into account additional shift of the dispersion due to change in refractive index. To perform the fit, we extracted the peaks in the measured extinction (as $1-$transmission) spectra for each k and used a standard least squares fitting procedure to minimize the difference between the eigenvalues of the matrix and the peaks extracted from the measurements. In the sample without electrodes (main text Fig. 1 and Fig. 2), the obtained values were $\Omega_{\text{R}} = 52$ meV and $s = -12$ meV. In the sample with electrodes (main text Fig. 3 and Fig. 4), the obtained values were $\Omega_{\text{R}} = 46$ meV and $s = -6$ meV.

Section S4. Pulsed laser excitation

PL measurements with fs-pulsed laser excitation are shown in Fig. S3. As illustrated in Fig. S3d, fs-pulsed excitation can result in a PL enhancement factor exceeding 100. Interestingly, the fs-pulsed excitation leads to emission from lower energy states along the dispersion branch compared

to continuous-wave excitation, close to the bound states in the continuum (BIC) mode of the lattice.[15?–18] Additionally, as shown in Fig. S3e, the emission linewidth is narrower (8 meV) than that observed in the transmission measurement at $k = 0$ (17 meV) in main text Fig. 2c-d. The observed red shift of emission might indicate a thermalization process, while the reduced linewidth could suggest the onset of lasing/polariton condensation, as has been seen in prior experiments with dye molecules.[11,19] However, further investigation is needed to confirm the origin of the observed features, which is beyond the scope of this study.

References

(1) Draine, B. T.; Flatau, P. J. Discrete-Dipole Approximation For Scattering Calculations. *Journal of the Optical Society of America A* **1994**, *11*, 1491–1499.

(2) Steshenko, S.; Capolino, F. In *Theory and Phenomena of Metamaterials*; Capolino, F., Ed.; CRC Press: Boca Raton, 2009; Chapter 8.

(3) Martikainen, J.-P.; Moilanen, A. J.; Törmä, P. Coupled dipole approximation across the -point in a finite-sized nanoparticle array. *Philosophical Transactions of the Royal Society A: Mathematical, Physical and Engineering Sciences* **2017**, *375*, 20160316.

(4) Meier, M.; Wokaun, A. Enhanced fields on large metal particles: dynamic depolarization. *Optics Letters* **1983**, *8*, 581–583.

(5) Johnson, P. B.; Christy, R. W. Optical Constants of the Noble Metals. *Physical Review B* **1972**, *6*, 4370–4379.

(6) Kravets, V. G.; Kabashin, A. V.; Barnes, W. L.; Grigorenko, A. N. Plasmonic Surface Lattice Resonances: A Review of Properties and Applications. *Chemical Reviews* **2018**, *118*, 5912–5951.

(7) Le-Van, Q.; Zoethout, E.; Geluk, E.-J.; Ramezani, M.; Berghuis, M.; Gómez Rivas, J. Enhanced Quality Factors of Surface Lattice Resonances in Plasmonic Arrays of Nanoparticles. *Advanced Optical Materials* **2019**, *7*, 1801451.

(8) Törmä, P.; Barnes, W. L. Strong Coupling between Surface Plasmon Polaritons and Emitters: A Review. *Reports on Progress in Physics* **2015**, *78*, 013901.

(9) Heilmann, R.; Väkeväinen, A. I.; Martikainen, J.-P.; Törmä, P. Strong coupling between organic dye molecules and lattice modes of a dielectric nanoparticle array. *Nanophotonics* **2020**, *9*, 267–276.

(10) Väkeväinen, A. I.; Moerland, R. J.; Rekola, H. T.; Eskelinen, A.-P.; Martikainen, J.-P.; Kim, D.-H.; Törmä, P. Plasmonic Surface Lattice Resonances at the Strong Coupling Regime. *Nano Letters* **2014**, *14*, 1721–1727.

(11) Väkeväinen, A. I.; Moilanen, A. J.; Nečada, M.; Hakala, T. K.; Daskalakis, K. S.; Törmä, P. Sub-picosecond thermalization dynamics in condensation of strongly coupled lattice plasmons. *Nature Communications* **2020**, *11*, 3139.

(12) Lee, B.; Liu, W.; Naylor, C. H.; Park, J.; Malek, S. C.; Berger, J. S.; Johnson, A. T. C.; Agarwal, R. Electrical Tuning of Exciton–Plasmon Polariton Coupling in Monolayer MoS2 Integrated with Plasmonic Nanoantenna Lattice. *Nano Letters* **2017**, *17*, 4541–4547.

(13) Wang, S.; Le-Van, Q.; Vaianella, F.; Maes, B.; Eizagirre Barker, S.; Godiksen, R. H.; Curto, A. G.; Gomez Rivas, J. Limits to Strong Coupling of Excitons in Multilayer WS2 with Collective Plasmonic Resonances. *ACS Photonics* **2019**, *6*, 286–293.

(14) Tabataba-Vakili, F.; Krelle, L.; Husel, L.; Nguyen, H. P. G.; Li, Z.; Bilgin, I.; Watanabe, K.; Taniguchi, T.; Högele, A. Metasurface of Strongly Coupled Excitons and Nanoplasmonic Arrays. *Nano Letters* **2024**, *24*, 10090–10097.

(15) Hakala, T. K.; Rekola, H. T.; Väkeväinen, A. I.; Martikainen, J.-P.; Nečada, M.; Moilanen, A. J.; Törmä, P. Lasing in Dark and Bright Modes of a Finite-Sized Plasmonic Lattice. *Nature Communications* **2017**, *8*, 13687.

(16) Heilmann, R.; Salerno, G.; Cuerda, J.; Hakala, T. K.; Törmä, P. Quasi-BIC Mode Lasing in a Quadrumer Plasmonic Lattice. *ACS Photonics* **2022**, *9*, 224–232.

(17) Mohamed, S.; Wang, J.; Rekola, H.; Heikkinen, J.; Asamoah, B.; Shi, L.; Hakala, T. K. Controlling Topology and Polarization State of Lasing Photonic Bound States in Continuum. *Laser & Photonics Reviews* **2022**, *16*, 2100574.

(18) Ardizzone, V. et al. Polariton Bose–Einstein condensate from a bound state in the continuum. *Nature* **2022**, *605*, 447–452.

(19) Hakala, T. K.; Moilanen, A. J.; Väkeväinen, A. I.; Guo, R.; Martikainen, J.-P.; Daskalakis, K. S.; Rekola, H. T.; Julku, A.; Törmä, P. Bose-Einstein Condensation in a Plasmonic Lattice. *Nature Physics* **2018**, *14*, 739.
